# Author response to: Comment on: Minimally invasive *versus* open pancreatic surgery: meta-analysis of randomized clinical trials

**DOI:** 10.1093/bjsopen/zrad087

**Published:** 2023-09-12

**Authors:** Matthias Pfister, Markus K Muller, Pascal Probst

**Affiliations:** Department of Surgery, Cantonal Hospital Thurgau, Frauenfeld, Switzerland; Department of Surgery and Transplantation, University Hospital Zurich, Zurich, Switzerland; Department of Surgery, Cantonal Hospital Thurgau, Frauenfeld, Switzerland; Department of Surgery, Cantonal Hospital Thurgau, Frauenfeld, Switzerland

*Editor*

The critical appraisal^1^ of the recently published meta-analysis of RCTs comparing minimally invasive with open pancreatic surgery^2^ is appreciated. Several important points were raised, and the authors would like to address them individually.

With regard to the importance of distinguishing between laparoscopic and robotic surgery, to the best of the authors’ knowledge, no data from RCTs comparing robotic, laparoscopic, and open pancreatic surgery separately are available^3^. Although it is agreed that robust data on the presumed advantages of robotic over laparoscopic surgery are urgently needed by the pancreatic surgical community to improve patient care, a distinction was not possible.

Given that learning curves represent a substantial source of bias in surgical literature, the systematic review reported centre and surgeon experience. Indeed, the authors strongly believe that reporting of learning curves, as well as surgeon and centre experience, is mandatory for data interpretation^4,5^.

While long-term oncological and quality-of-life outcomes are of paramount importance, no such data were available from RCTs regarding the robotic approach. Once these data are published, it will be of extreme interest to carry out a meta-analysis. The lack of additional specific robotic pancreatic surgery outcomes was highlighted. However, while the proposed outcomes are certainly of great interest, in the literature, data were only available from prospective and retrospective studies.
